# High-Density Livestock Production and Molecularly Characterized MRSA Infections in Pennsylvania

**DOI:** 10.1289/ehp.1307370

**Published:** 2014-02-07

**Authors:** Joan A. Casey, Bo Shopsin, Sara E. Cosgrove, Keeve E. Nachman, Frank C. Curriero, Hannah R. Rose, Brian S. Schwartz

**Affiliations:** 1 Department of Environmental Health Sciences, Johns Hopkins Bloomberg School of Public Health, Baltimore, Maryland, USA; 2 Center for a Livable Future, Johns Hopkins University, Baltimore, Maryland, USA; 3 Department of Medicine, New York University School of Medicine, New York, New York, USA; 4 Division of Infectious Disease, Johns Hopkins School of Medicine, Baltimore, Maryland, USA; 5 Department of Biostatistics, Johns Hopkins Bloomberg School of Public Health, Baltimore, Maryland, USA; 6 Department of Medicine, Johns Hopkins School of Medicine, Baltimore, Maryland, USA; 7 Center for Health Research, Geisinger Health System, Danville, Pennsylvania, USA

## Abstract

Background: European studies suggest that living near high-density livestock production increases the risk of sequence type (ST) 398 methicillin-resistant *Staphylococcus aureus* (MRSA) colonization. To our knowledge, no studies have evaluated associations between livestock production and human infection by other strain types.

Objectives: We evaluated associations between MRSA molecular subgroups and high-density livestock production.

Methods: We conducted a yearlong 2012 prospective study on a stratified random sample of patients with culture-confirmed MRSA infection; we oversampled patients from the Geisinger Health System with exposure to high-density livestock production in Pennsylvania. Isolates were characterized using *S. aureus* protein A (*spa*) typing and detection of Panton-Valentine leukocidin (PVL) and *scn* genes. We compared patients with one of two specific MRSA strains with patients with all other strains of MRSA isolates, using logistic regression that accounted for the sampling design, for two different exposure models: one based on the location of the animals (livestock model) and the other on crop field application of manure (crop field model).

Results: Of 196 MRSA isolates, we identified 30 *spa* types, 47 PVL-negative and 15 *scn*-negative isolates, and no ST398 MRSA. Compared with quartiles 1–3 combined, the highest quartiles of swine livestock and dairy/veal crop field exposures were positively associated with community-onset-PVL-negative MRSA (CO-PVL-negative MRSA vs. all other MRSA), with adjusted odds ratios of 4.24 (95% CI: 1.60, 11.25) and 4.88 (95% CI: 1.40, 17.00), respectively. The association with CO-PVL-negative MRSA infection increased across quartiles of dairy/veal livestock exposure (trend *p* = 0.05).

Conclusions: Our findings suggest that other MRSA strains, beyond ST398, may be involved in livestock-associated MRSA infection in the United States.

Citation: Casey JA, Shopsin B, Cosgrove SE, Nachman KE, Curriero FC, Rose HR, Schwartz BS. 2014. High-density livestock production and molecularly characterized MRSA infections in Pennsylvania. Environ Health Perspect 122:464–470; http://dx.doi.org/10.1289/ehp.1307370

## Introduction

Over the past decade, the incidence of community-associated methicillin-resistant *Staphylococcus aureus* (*S. aureus*) (CA-MRSA) infection has increased in the United States (Dukic et al. 2013). These CA-MRSA infections cost third-party payers between $478 and $2,200 million annually (Lee et al. 2013). Beginning in the mid-2000s, European research suggested that a portion of the increased incidence of CA-MRSA might be attributable to high-density livestock production because studies had isolated the same MRSA strains from infected farmers and their livestock (Harrison et al. 2013; Hartmeyer et al. 2010). In Europe, multilocus sequence type (ST) 398 has been the most common colonizer of livestock, specifically swine, and swine farmers (Graveland et al. 2010; Lewis et al. 2008). This association has led many to refer to ST398 as livestock-associated MRSA (LA-MRSA) (Price et al. 2012). Importantly, pathways for community transmission have been identified, with MRSA isolated from the air and soil at least 150 m from swine facilities (Gibbs et al. 2006; Schulz et al. 2012) and from meat processing and consumption (Molla et al. 2012; Waters et al. 2011).

To our knowledge, no North American studies have evaluated residence in rural communities as a risk factor for MRSA infection of specific molecular types. Four studies of colonization in farmers have disparate findings. The first reported ST398 as the only type colonizing swine and farmers (Smith et al. 2009); the second found mainly ST398, but also ST5 (Khanna et al. 2008); and two more recent studies identified ST5 [t002 by *S. aureus* protein A (*spa*) typing (Monecke et al. 2011)] as the primary colonizer of swine and of veterinary students visiting farms (Frana et al. 2013; Molla et al. 2012). In the United States, ST5 has been considered to be a health care–associated MRSA clone, but it has begun to appear in the community in persons without health care risk factors (Klevens et al. 2006). Nearly all MRSA isolates associated with livestock colonization lack the genes encoding Panton-Valentine leukocidin (PVL) (Smith et al. 2009; Sunde et al. 2011). Finally, recent studies have reported that *scn,* the gene encoding staphylococcal complement inhibitor (SCIN), is often absent in MRSA strains that colonize livestock (McCarthy et al. 2011; Sung et al. 2008; Verkaik et al. 2011).

In a previous study in Pennsylvania using electronic health records from 2005 through 2010 from a large health care system, we reported associations between CA-MRSA infection and residential proximity to high-density livestock operations and the crop fields to which manure was applied (Casey et al. 2013b). Due to the retrospective study design, we were unable to obtain MRSA isolates from patients. The objectives of the present study were to *a*) prospectively collect MRSA isolates from patients residing in communities with and without high-density livestock production, *b*) characterize these isolates by *spa* typing and polymerase chain reaction (PCR) for the presence of *lukF-lukS* genes (encoding PVL) and for the *scn* gene, and *c*) assess associations of high-density livestock production with the molecular subgroups.

## Methods

**Figure 1:**
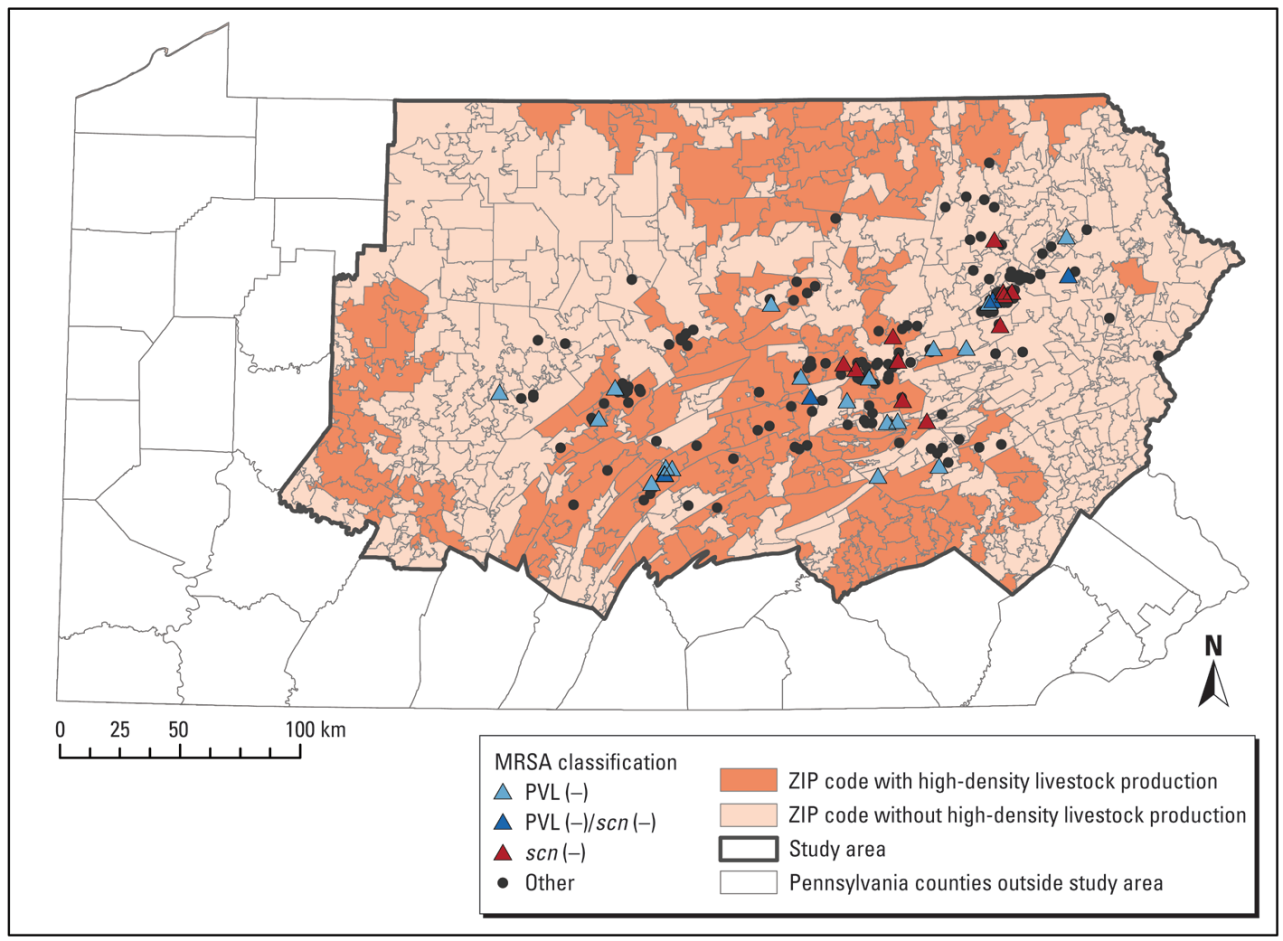
High-density livestock production and MRSA infection, Geisinger Health Care System, Pennsylvania.

*Setting, study design, and participants*. The study area was a 38-county region of central and northeast Pennsylvania with 3.8 million inhabitants in which Geisinger Health System provides primary care services from 41 community practice clinics (Figure 1). The primary care population is representative of the region’s population (Casey et al. 2013b). We compared patients infected with two specific MRSA strains to patients infected with all other strains as described below. Institutional review boards at the Geisinger Health System and the Johns Hopkins Bloomberg School of Public Health approved the study and waived informed consent.

Patients with MRSA infection identified by culture between 1 January 2012 and 31 December 2012, who had a Geisinger primary care provider, were eligible for inclusion. The cases were stratified as community-onset or hospital-onset and as residing in an area of high-density livestock production or not. Infections were classified as community-onset (CO) infections if the patient had a positive culture collected in the outpatient setting or ≤ 2 days after hospital admission. Infections were classified as hospital-onset (HO) if the patient had a positive culture collected > 2 days after hospital admission (David and Daum 2010). Onset date was defined as the date the culture was ordered. Patients living in a ZIP code with a high-density livestock operation or a crop field to which manure from such an operation was applied were defined as residing in an area of high-density livestock production, as described in detail below. Using these case definitions, we completed a random stratified sample (a sample was evaluated because of budgetary limitations) to select cases into four *a priori* categories (Table 1). We over-selected patients who resided in areas of high-density livestock production to improve the power to detect associations.

**Table 1:** Patients identified and included in the present analysis according to MRSA subtype and exposure in their residential ZIP code.

Group	High-density livestock production	No high-density livestock production	Total
CO-MRSA
Patients included [*n* (%)]	70 (42.2)	96 (57.8)	166
Total patients [*n* (%)]	321 (32.5)	667 (67.5)	988
Sample weight^*a*^	4.59	6.95
HO-MRSA
Patients included [*n* (%)]	11 (36.7)	19 (63.3)	30
Total patients [*n* (%)]	54 (22.5)	186 (77.5)	240
Sample weight^*a*^	4.91	9.79
Total patients included (*n*)	81	115	196
Total patients identified (*n*)	375	853	1,128
Abbreviations: CO, community-onset; HO, hospital-onset. ^***a***^Sample weight was calculated as the total number of patients identified in each stratum divided by the number of patients sampled from that stratum for the present analysis.			

*Patient data collection*. MRSA isolates (one per patient) collected in clinical practice settings were sent to a central laboratory, grown on conventional blood agar plates, and stored at 4°C. A research assistant selected appropriate isolates and obtained samples for transport using sterile swab applicators (BBL Cultureswabs; Becton Dickinson, Franklin Lakes, NJ). A total of 203 unique isolates collected from sites of infection were sent unrefrigerated to New York University Langone Medical Center for genotypic testing, where isolates were subcultured onto agar plates and incubated overnight at 37°C. DNA was then extracted by mechanical lysis. We obtained information on patient demographics and inpatient, outpatient, and emergency department encounters, procedures, medication orders, and laboratory data from the electronic health records. Antimicrobial resistance testing on clindamycin, erythromycin, gentamicin, rifampin, tetracycline, trimethoprim–sulfamethoxazole, and vancomycin was also available from the electronic health records. Multidrug resistance was reported as resistance to three or more unique antimicrobials.

*Genotyping*. DNA sequence analysis of the protein A gene variable repeat region (*spa* typing) was completed using the primers TIGR-F (5´-GCCA​AAGC​GCTA​ACCT​TTTA​-3´) and TIGR-R (5´-TCCA​GCTA​ATAA​CGCT​GCAC​-3´) (Shopsin et al. 1999). Spa types were assigned a StaphType (e.g., t008, t002) using the Ridom SpaServer database (http://www.spaserver.ridom.de). We subsequently mapped *spa* types to clonal complex (CC) categories (CC5 or CC5-like, CC8 or CC8-like, or other) using the Ridom SpaServer *spa* to multilocus sequence type (MLST) database (http://spa.ridom.de/mlst.shtml). We also checked for the presence of PVL genes using *S. aureus* ATCC 49775 (ATCC, Manassas, VA) as a reference strain and primers as previously described (Lina et al. 1999). The presence of *scn* was determined by an additional PCR (Sung et al. 2008).

Earlier literature from northern Europe suggested that ST398 would be a cause of human infection in those exposed to high-density livestock production (Hartmeyer et al. 2010; Wulf et al. 2012); however, we did not identify any ST398 infections. Therefore, before beginning statistical analysis, we identified two other strains of interest. Although most CA-MRSA contains PVL genes (David and Daum 2010), most MRSA (both ST5 and ST398) isolated from humans and animals with exposure to high-density livestock production in North America has been PVL-negative (Frana et al. 2013; Price et al. 2012; Tattevin et al. 2012). Therefore, we selected community-onset PVL-negative (CO-PVL-negative) MRSA isolates as a unique group with potential ties to high-density livestock production. We created a second molecular subgroup based on recent evidence that only about 25% of animal *S. aureus* isolates carry the *scn* gene, whereas human isolates carry it about 90% of the time (Sung et al. 2008; Verkaik et al. 2011). To complete the analysis, we created two case groups—CO-PVL-negative MRSA and *scn*-negative MRSA—and compared these groups to patients with all other MRSA types.

*Geospatial estimates of high-density livestock exposure*. Pennsylvania Act 38 of 2005 (2005) requires nutrient management plans (NMPs) to regulate concentrated animal operations, operations where livestock density exceeds two animal equivalent units (AEUs; 1,000 pounds of animal weight) per acre and where the total number of AEUs exceeds eight. NMPs detail appropriate manure handling, storage, and land application to crop fields, both on-site where the animals are raised and off-site. NMPs also contain data on operation location and acreage and livestock type. In addition, the Pennsylvania Department of Environmental Protection requires NMPs from the largest operations called concentrated animal feeding operations (> 1,000 AEUs, or 301–1,000 AEUs and a concentrated animal operation, or per federal regulation) (U.S. Environmental Protection Agency 2012). For our analysis, we defined a high-density livestock operation as *a*) > 2 AEUs per acre and > 8 AEUs total, or *b*) > 300 AEUs total.

We used ArcGIS (version 10.0; Esri, Redlands, CA) to geocode livestock operations, treated crop fields, and patients at their home address. Although patients were sampled using presence of high-density livestock production in their ZIP code, this criterion was not used to generate exposure metrics. We created two exposure metrics for livestock operations (swine or dairy/veal):

Exposure for patient *j* = Σ*^^n^^_i_*_= 1_(*a_i_*/*d_ij_*^2^), [1]

where *n* is the number of operations, *a_i_* is the AEUs of livestock at operation *i*, and *d_ij_*^2^ is the squared distance (in meters) between operation *i* and patient *j*. In addition, we created two metrics for exposure to treated crop fields (swine or dairy/veal manure applications) during the season of infection:

Exposure for patient *j* = Σ*^^n^^_k_*_= 1_(*c_k_*/*d_kj_*^2^), [2]

where *n* is the number of treated crop fields, *c_k_* is the concentration of manure (gallons per square meter) applied to field *k* during the season of diagnosis, and *d_kj_*^2^ is the squared distance (in meters) between field centroid *k* and patient *j* (Casey et al. 2013b). We modeled the exposure variables as quartiles to allow for nonlinear associations and for ease of interpretation.

*Statistical methods*. A multivariable logistic regression model was used to estimate associations between exposures and outcomes (i.e., CO-PVL-negative MRSA or *scn*-negative MRSA vs. all other MRSA strains combined). By study design, we oversampled cases living in ZIP codes with high-density livestock production. To account for this sampling design while obtaining unbiased regression estimates and robust standard errors, each participant was assigned a sample weight equal to the total number of MRSA patients identified in a stratum/total number included in the present analysis (Table 1), a measure of the number of patients represented by each sampled individual (Vittinghoff et al. 2005). We used the *p*-value from Fisher’s exact test to compare molecular profiles of the two subgroups of interest to all other MRSA isolates (Table 2).

**Table 2:** Onset location, Ridom *spa* types, and PVL presence by MRSA subgroup [*n* (%)].

Characteristic	CO-PVL-negative^*a*^ (*n *= 23)	*scn*-negative^*a*^ (*n *= 15)	All other MRSA (*n *= 162)
CO-MRSA	23 (100)^*b*^	12 (80.0)	135 (83.3)
CC5 or CC5-like
t002	9 (39.1)^*b*^	1 (6.7)	17 (10.5)
t010	2 (8.7)^*b*^	0	0
t045	0	1 (6.7)	1 (0.6)
t062	1 (4.3)	0	0
t088	3 (13.0)^*b*^	1 (6.7)	0
t105	1 (4.3)	1 (6.7)	2 (1.2)
t306	1 (4.3)	0	0
t6614	1 (4.3)	1 (6.7)	0
Other^*c*^	0	0	3 (1.8)
CC8 or CC8-like
t008	0^*b*^	7 (46.7)	115 (71.0)
t024	0	1 (6.7)	6 (3.7)
t068	0	0	2 (1.2)
t121	0	1 (6.7)	2 (1.2)
t622	0	0	3 (1.9)
Other^*d*^	0	0	7 (4.3)
Other CC
t125	1 (4.3)	1 (6.7)	0
t948	1 (4.3)	0	0
t9964	1 (4.3)	0	0
t11970	1 (4.3)	0	0
Other^*e*^	0	0	3 (1.9)
Novel^*f*^	1 (4.3)	0	1 (0.6)
PVL-positive	0^*b*^	9 (60.0)^*b*^	140 (86.4)
Percentages do not add to 100 due to rounding. ^***a***^Four patients were in both the CO-PVL-negative group and the *scn*-negative group. ^***b***^*p*-Value < 0.05 comparing two subgroups of interest to all other MRSA using Fisher’s exact test. ^***c***^One each of t437, t539, and t85. ^***d***^One each of t064, t206, t211, t304, t681, t692, t1610. ^***e***^One each of t216, t316, t11971. ^***f***^Novel *spa* type.			

Models were constructed with attention to the small sample size. Our primary models compared the highest quartile of exposure to livestock or treated crop fields to all other quartiles combined. In addition, we modeled quartiles of exposure as an ordinal variable (0, 1, 2, 3) and used the *p*-value of the resulting coefficient as a test of linear trend. For CO-PVL-negative MRSA, we also estimated associations with individual quartiles of exposure relative to the lowest quartile, but numbers of observations were too small to run comparable models for *scn*-negative MRSA. Similarly, adjustment for confounders selected based on *a priori* information (Casey et al. 2013a, 2013b) was limited to models of CO-PVL-negative MRSA. Specifically, we adjusted for sex, age (continuous), ever-smoking status, season of infection [winter (December–February) vs. all other seasons combined], physician order for an antibiotic in the 365 to 14 days preceding diagnosis, and ever-receiving Medical Assistance as health insurance [as a surrogate for low individual socioeconomic status (Bratu et al. 2006; Casey et al. 2013b)]. We were unable to adjust models for race/ethnicity and to isolate sources because data were off-support (Oakes 2006). We did not adjust for location of onset because location was part of the CO-PVL-negative MRSA definition. Statistical analyses were performed using Stata 11.2 (StataCorp, College Station, TX) using the svy commands for weighted regression and R version 3.0.0 (R Foundation for Statistical Computing, Vienna, Austria).

## Results

*Study population characteristics*. From 1 January to 31 December 2012 we collected data on 203 MRSA infections (from a pool of 1,128 patients with MRSA infection) from patients in 20 counties in Pennsylvania. The analysis included 196 isolates (Figure 1) after exclusion of isolates lacking the *mecA* gene (*n* = 4) and patients who could not be geocoded or resided outside the study area (*n* = 3). We identified 30 unique Ridom *spa* types, 47 PVL-negative isolates and 15 *scn*-negative isolates, and no ST398 MRSA (Table 2). Four isolates were both CO-PVL-negative and *scn*-negative. Patients with CO-PVL-negative MRSA (*n* = 23) as well as patients with *scn*-negative MRSA (*n* = 15) were older than patients in the all other MRSA group (*n* = 162) (Table 3). Patients with the two MRSA strains of interest were less likely to reside in cities than were patients with all other MRSA strains (Table 3). Patients in the third and fourth quartiles of swine livestock exposure were more likely than those in the first and second quartiles to be diagnosed in the autumn (Table 3). Approximately 95% of patients with MRSA onset in the winter fell into the first or second quartile of swine and dairy/veal crop field exposure (data not shown).

**Table 3:** Patient demographic and clinical characteristics of MRSA subgroups by swine livestock exposure quartile^*a*^ [data are *n* (%) unless otherwise indicated]

Characteristic	CO-PVL-negative (*n *= 23)^*b*^	*scn*-negative (*n *= 15)^*b*^	All other MRSA (*n *= 162)	Swine (quartile)			
				1	2	3	4
Sex, male	15 (65.2)	11 (73.3)	85 (52.5)	28 (26.2)	25 (23.4)	24 (22.4)	30 (28.0)
Age at infection or visit [median (interquartile range)] (years)	54.2 (32.3–75.2)	52.8 (14.6–75.1)	32.8 (14.4–55.2)	46.0 (17.3–62.4)	33.6 (22.5–54.7)	31.1 (9.8–51.7)	37.5 (13.6–59.1)
Race/ethnicity^*c*^
Non-Hispanic white	22 (95.7)	15 (100)	155 (95.7)	44 (23.4)	49 (26.1)	48 (25.5)	47 (25.0)
Non-Hispanic black	1 (4.4)	0	6 (3.7)	5 (71.4)	0	0	2 (28.6)
Other	0	0	1 (0.6)	0	0	1 (100)	0
Never-smoker	16 (69.6)	10 (66.7)	139 (85.8)	39 (23.9)	43 (26.4)	40 (24.5)	41 (25.2)
Season of onset
Winter	7 (30.4)	3 (20.0)	27 (16.7)	12 (34.3)	7 (20.0)	7 (20.0)	9 (25.7)
Spring	5 (21.7)	4 (26.7)	37 (22.8)	16 (34.8)	13 (28.3)	9 (19.6)	8 (17.4)
Summer	7 (30.4)	5 (33.3)	39 (24.1)	10 (20.4)	16 (32.7)	12 (24.5)	11 (22.5)
Autumn	4 (17.4)	3 (20.0)	59 (36.4)	11 (16.7)	13 (19.7)	21 (31.8)	21 (31.8)
Antibiotic prescription in previous year	5 (21.7)	6 (40.0)	65 (40.1)	21 (27.6)	20 (26.3)	15 (19.7)	20 (26.3)
Source
Skin/soft tissue	19 (82.6)	11 (73.3)	141 (87.0)	36 (25.9)	39 (28.1)	28 (20.1)	36 (25.9)
Respiratory	0	2 (13.3)	15 (9.3)	6 (35.3)	4 (23.5)	4 (23.5)	3 (17.7)
Bone	2 (8.7)	1 (6.7)	2 (1.2)	1 (25.0)	0	2 (50.0)	1 (25.0)
Other	2 (8.7)	1 (6.7)	4 (2.5)	0	3 (42.9)	2 (28.6)	2 (28.6)
Antibiotic resistance^*d*^
Clindamycin	12 (52.2)	2 (13.3)	32 (19.8)	10 (21.7)	11 (23.9)	13 (28.3)	12 (26.1)
Erythromycin	18 (78.3)	12 (80.0)	149 (92.0)	43 (24.4)	46 (26.1)	46 (26.1)	41 (23.3)
Tetracycline	4 (17.4)	1 (6.7)	4 (2.5)	2 (25.0)	2 (25.0)	2 (25.0)	2 (25.0)
TMP-SMZ	1 (4.4)	1 (6.7)	2 (1.2)	2 (66.7)	1 (33.3)	0	0
Multidrug^*e*^	14 (60.9)	3 (20.0)	33 (20.4)	12 (24.5)	11 (22.5)	14 (28.6)	12 (24.5)
Community type
City	1 (4.4)	0	23 (14.2)	11 (45.8)	3 (12.5)	9 (37.5)	1 (4.2)
Borough	11 (47.8)	5 (33.3)	44 (27.2)	15 (25.9)	15 (25.9)	8 (13.8)	20 (34.5)
Township	11 (47.8)	10 (66.7)	95 (58.6)	23 (20.2)	31 (27.2)	32 (28.1)	28 (24.6)
Medical Assistance
Never received	15 (65.2)	10 (66.7)	127 (78.4)	35 (23.3)	37 (24.7)	41 (27.3)	37 (24.7)
Location of onset
Community	27 (100)	15 (83.3)	135 (83.3)	41 (24.7)	43 (25.9)	40 (24.1)	42 (25.3)
Hospital	0	3 (16.7)	27 (16.7)	8 (26.7)	6 (20.0)	9 (30.0)	7 (23.3)
No. of patients with high-density livestock production in their residential ZIP code	9 (39.1)	7 (46.7)	67 (41.4)	1 (1.2)	17 (21.0)	25 (30.9)	38 (46.9)
Abbreviations: previous year, 365 to 14 days before infection; TMP-SMZ, trimethoprim–­ sulfamethoxazole.^***a***^Quartile 1: swine livestock exposure < 6.20 AEU/km^2^; quartile 2: 6.20–16.00 AEU/km^2^; quartile 3: 16.01–33.40 AEU/km^2^; quartile 4: > 33.41 AEU/km^2^. ^***b***^Four patients were in both the CO-PVL-negative group and the *scn*-negative group. ^***c***^Race/ethnicity was missing for two members of the all other MRSA isolates comparison group. ^***d***^Resistance to gentamicin was observed in one CO-PVL-negative isolate, and intermediate resistance to rifampin was observed in one all other MRSA isolates. ^***e***^Resistance to three or more unique antimicrobials.							

*Molecular testing*. We identified 30 *spa* types, but 2 predominated: t008 [*n* = 122 (62.2%)] and t002 [*n* = 27 (13.8%)] (Table 2). Nearly all t008, *n* = 116 (95.1%), was community-onset, whereas the majority of t002, *n* = 17 (63.0%), was hospital-onset. Isolates lacking *scn* (*n* = 15) were distributed across multiple *spa* types, and 80% (*n* = 12) were community-onset. CO-PVL-negative isolates were mostly *spa* types associated with CC5.

*Antimicrobial susceptibility*. CO-PVL-negative strains were more often resistant to antibiotics commonly used to treat MRSA infection (i.e., clindamycin, tetracycline, and trimethoprim–sulfamethoxazole) than all other CO-PVL-positive MRSA strains. The 30 hospital-onset (HO)-MRSA isolates were resistant to more antibiotics compared with CO-MRSA: clindamycin (83.3% vs. 12.7%), tetracycline (6.7% vs. 3.6%), trimethoprim–sulfamethoxazole (3.3% vs. 1.2%), and multidrug (83.3% vs. 14.5%). Resistance patterns did not appear to be associated with agriculture exposure variables, but tetracycline resistance in a small number of isolates (*n* = 4) was associated with infection by CO-PVL-negative MRSA (*p* = 0.02).

**Figure 2:**
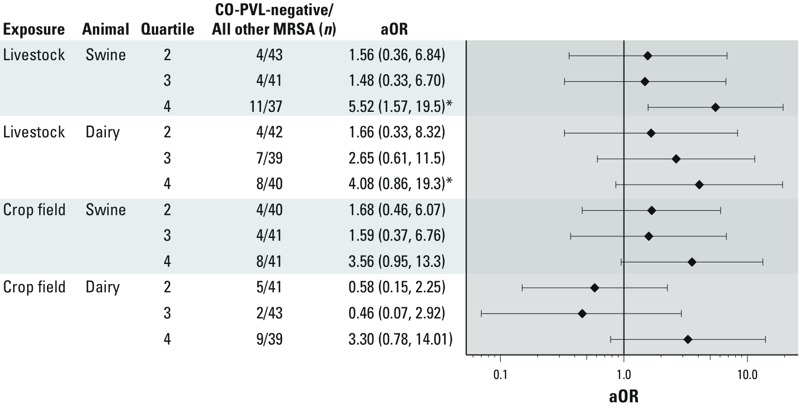
Association of swine and dairy/veal seasonal crop field quartiles and livestock quartiles with CO-PVL-negative MRSA status from adjusted models. Adjusted for sex, age, ever-smoking status, antibiotic order in the 365 to 14 days before infection, season, and receipt of Medical Assistance. *p-Value for linear trend ≤ 0.05 (quartiles included as a single variable with values 1, 2, 3, and 4).

*Associations with livestock and crop field manure exposure*. High-density livestock production was not associated with *scn*-negative MRSA infection in unadjusted analysis (Table 4). Swine livestock exposure was associated with CO-PVL-negative MRSA in unadjusted and adjusted analysis (Table 4), which appeared driven by the association in the fourth quartile of exposure (Figure 2). There was a positive but nonsignificant association with the dichotomized dairy/veal livestock exposure variable [odds ratio (OR) = 2.42; 95% CI: 0.85, 6.88]. There was a trend (*p* = 0.05) of increasing odds of CO-PVL-negative MRSA across quartiles of dairy/veal livestock exposure; however, all ORs were increasingly imprecise with higher exposure for individual versus lowest quartile exposures (Figure 2). Those in the highest quartile of swine crop field exposure had an OR of 2.38 (95% CI: 0.78, 7.28) of being a CO-PVL-negative MRSA case compared with those in quartiles 1–3 (Table 4). Dichotomous dairy/veal crop field exposure was associated with CO-PVL-negative MRSA in adjusted analyses (OR = 4.88; 95% CI: 1.40, 17.00) (Table 4). Neither crop field variable evidenced a statistically significant trend across quartiles (Figure 2). Associations strengthened slightly as we added sex, age, ever-smoking, antibiotic order, and Medical Assistance to the models. Season of onset was a strong confounder in the crop field exposure models [e.g., the unadjusted OR of 0.79 (95% CI: 0.20, 3.09) increased to 2.38 (95% CI: 0.78, 7.28) (Table 4) for the swine crop field model, primarily due to the inclusion of season as a variable].

**Table 4:** Unadjusted and adjusted^*a*^ associations of dichotomous exposures with CO-PVL-negative MRSA and *scn*-negative MRSA compared with all other MRSA (n/N).

Exposure		CO-PVL-negative^*b*^			*scn*-negative^*b*^	
		All other MRSA (*n/N*)	Unadjusted OR (95% CI)	Adjusted OR^*c*^ (95% CI)	All other MRSA (*n/N*)	Unadjusted OR (95% CI)
Livestock	
Swine (quartile)	1, 2, 3	12/124	1.0	1.0	12/125	1.0
	4^*d*^	11/37	3.55 (1.41, 8.94)	4.24 (1.60, 11.25)	3/37	0.79 (0.20, 3.09)
Dairy/veal (quartile)	1, 2, 3	15/122	1.0	1.0	13/122	1.0
	4^*e*^	8/40	1.57 (0.61, 4.05)	2.42 (0.85, 6.88)	2/40	0.47 (0.10, 2.24)
Treated crop field	
Swine (quartile)	1, 2, 3	15/121	1.0	1.0	14/121	1.0
	4^*f*^	8/41	1.55 (0.59, 4.05)	2.38 (0.78, 7.28)	1/41	0.18 (0.02, 1.43)
Dairy/veal (quartile)	1, 2, 3	14/123	1.0	1.0	13/123	1.0
	4^*g*^	9/39	2.28 (0.89, 5.83)	4.88 (1.40, 17.00)	2/39	0.46 (0.10, 2.20)

## Discussion

This analysis compared patients infected by two specific MRSA strains to patients infected by all other MRSA strains and found a number of associations with high-density livestock production. The livestock model incorporated AEUs at the farming operation and distance to the patient residence; whereas the crop field model incorporated manure volume, crop field area, and distance to the patient residence. Although adjusted estimates were imprecise because of small sample numbers, each exposure variable was associated with increased odds of CO-PVL-negative MRSA infection, and three of four were statistically significant. Specifically, there were increased odds comparing the fourth quartiles to the other three quartiles of both swine livestock exposure and dairy/veal crop field exposure. There was also a significant trend of increasing odds across the four quartiles for dairy/veal livestock exposure. For the fourth exposure variable, swine crop field, the OR in the fourth quartile versus the other three was higher, but not statistically significant. Unexpectedly, we did not identify any patients with MRSA infection due to ST398, the MRSA subtype that previously has been associated with livestock operations, mainly based on studies of colonization in Europe. This raises two issues. First, to understand risk factors for MRSA infection, we need to study MRSA infection rather than colonization. Second, although ST398 may be prevalent among swine and swine farmers in the United States, other strains found on farms may actually be more important for infection.

When we did not identify any infections due to ST398, we evaluated associations of livestock production with two other MRSA strains compared with all other MRSA strains. To our knowledge, only one U.S. study has reported PVL-positive MSRA isolated from swine (Osadebe et al. 2013). Associations with CO-PVL-negative MRSA were of interest because many highly successful human epidemic MRSA clones, like CC5, do not contain PVL genes and have recently been isolated from swine and swine farmers in the United States (Frana et al. 2013; Molla et al. 2012; Osadebe et al. 2013). We observed eight tetracycline-resistant isolates, four of which were CO-PVL-negative MRSA. This resistance could plausibly be a marker of high-density livestock production as tetracycline is the antibiotic most commonly sold for use in U.S. farm animals, and isolates resistant to tetracycline have been observed in workers at industrial livestock operations (Food and Drug Administration 2010; Rinsky et al. 2013). In addition, only 6.7% of HO-MRSA was resistant to tetracycline, suggesting that the association of CO-PVL-negative MRSA is not likely attributable to the health care system. Likewise, we selected *scn*-negative MRSA strains because we expected MRSA associated with livestock production to lack *scn* (McCarthy et al. 2011; Sung et al. 2008; Verkaik et al. 2011). Previous work showing selection against *scn* in animal hosts dealt primarily with ST398 (McCarthy et al. 2011), a strain of MRSA already adapted to animals (Price et al. 2012). Because the *scn* gene produces a protein that hinders phagocytosis of *S. aureus* by human neutrophils (Sung et al. 2008), this work might not translate directly to human-adapted strains of MRSA (e.g., ST5 or ST8), especially in the study of infection rather than colonization.

A few U.S. studies have molecularly characterized MRSA isolated from agricultural workers (Frana et al. 2013; Molla et al. 2012; Osadebe et al. 2013; Smith et al. 2009, 2013). Because those studies only described MRSA carriage in persons with direct contact with swine, they may have low utility regarding inference about MRSA infection in the community. Two studies reported transient carriage (< 24 hours) after short-term exposure to pigs and veal calves (Frana et al. 2013; van Cleef et al. 2011). Even if carriage persists after exposure, and the risk of infection increases, infection is not assured (Davis et al. 2004).

The body of literature on MRSA carriage is also relevant to the absence of ST398 among our MRSA isolates. Although there is ample evidence that those in contact with livestock have increased prevalence of ST398 MRSA colonization (Graveland et al. 2010; Smith et al. 2009; Voss et al. 2005), data suggest that ST398 MRSA infection is rare (Golding et al. 2010; Salmenlinna et al. 2010; Wulf et al. 2012). Strains of MRSA adapted primarily to animals (e.g., ST398) may have a diminished ability to infect humans (Graveland et al. 2011; Price et al. 2012). A recent Italian study reported a much greater proportion of ST398 among MRSA-colonized patients compared with those with a MRSA infection where ST8 was most prevalent (Monaco et al. 2013).

Confounding by occupation or socioeconomic status may have impacted our associations. We could not obtain patient occupation from the electronic health records and, therefore, cannot exclude the possibility that some CO-PVL-MRSA cases were occupational versus community acquired. We adjusted for Medical Assistance, a surrogate for low socioeconomic status that was associated with MRSA infection in our previous study (Casey et al. 2013b), but we acknowledge that there may be residual confounding due to socioeconomic status. Since the CO-PVL-negative MRSA group had several characteristics of health care–associated MRSA [(HA-MRSA), e.g., older age, antibiotic resistance, lack of PVL genes, many *spa* type t002] the association with high-density livestock production could have arisen if HA-MRSA was more common in rural areas. However, in a previous study conducted in this region, HA-MRSA was not more common in rural areas (Casey et al. 2013b). Also, HA-MRSA in the present analysis defined by common epidemiologic criteria (Morrison et al. 2006) was not associated with high-density livestock production (data not shown). We adjusted models for season of onset because, as expected, MRSA patients with onset in the winter had low exposure to treated crop fields (manure application is restricted in the winter).

The novel observations in this study require replication. We completed analysis on a limited number of patients with a MRSA infection and, although we incorporated several aspects of operations and crop fields into our individual-level exposure estimates, we did not collect environmental samples. Finally, we could not calculate incidence rates because we included only patients with MRSA infection.

## Conclusions

To our knowledge, no previous studies in the United States have evaluated associations between patients infected with specific MRSA strains and high-density livestock production. We identified associations of CO-PVL-negative MRSA with swine and dairy/veal livestock operations and application of dairy/veal manure to crop fields. Our findings were based on small numbers of observations and indirect estimates of exposure, but if confirmed, have important implications for the role of livestock operations in the MRSA epidemic, the different roles of colonization and infection studies, and the identification of other MRSA strains that may be arising from high-density livestock production. To help estimate the public health burden that high-density livestock production may place on the U.S. health care system, future studies should use MRSA infection, along with colonization, as their outcomes of interest. Our study also indicates the need to look beyond MRSA ST398 when investigating MRSA infection associated with high-density livestock production in the United States.

## References

[r1] Bratu S Landman D Gupta J Trehan M Panwar M Quale J 2006 A population-based study examining the emergence of community-associated methicillin-resistant *Staphylococcus aureus* USA300 in New York City. Ann Clin Microbiol Antimicrob 5 29; 10.1186/1476-0711-5-29 17137512 PMC1693566

[r2] Casey JA, Cosgrove SE, Stewart WF, Pollak J, Schwartz BS (2013a). A population-based study of the epidemiology and clinical features of methicillin-resistant *Staphylococcus aureus* infection in Pennsylvania, 2001-2010.. Epidemiol Infect.

[r3] Casey JA Curriero FC Cosgrove SE Nachman KE Schwartz BS 2013b High-density livestock operations, crop field application of manure, and risk of community-associated methicillin-resistant *Staphylococcus aureus* infection in Pennsylvania. JAMA Intern Med 173 1980 1990; 10.1001/jamainternmed.2013.10408 24043228 PMC4372690

[r4] David MZ, Daum RS (2010). Community-associated methicillin-resistant *Staphylococcus aureus*: epidemiology and clinical consequences of an emerging epidemic.. Clin Microbiol Rev.

[r5] Davis KA, Stewart JJ, Crouch HK, Florez CE, Hospenthal DR (2004). Methicillin-resistant *Staphylococcus aureus* (MRSA) nares colonization at hospital admission and its effect on subsequent MRSA infection.. Clin Infect Dis.

[r6] Dukic VM Lauderdale DS Wilder J Daum RS David MZ 2013 Epidemics of community-associated methicillin-resistant *Staphylococcus aureus* in the United States: a meta-analysis. PLoS One 8 1 e52722; 10.1371/journal.pone.0052722 23300988 PMC3534721

[r7] Food and Drug Administration. (2010). Summary Report on Antimicrobials Sold or Distributed for Use in Food-Producing Animals.. http://www.fda.gov/downloads/ForIndustry/UserFees/AnimalDrugUserFeeActADUFA/UCM277657.pdf.

[r8] Frana TS Beahm AR Hanson BM Kinyon JM Layman LL Karriker LA 2013 Isolation and characterization of methicillin-resistant *Staphylococcus aureus* from pork farms and visiting veterinary students. PLoS One 8 1 e53738; 10.1371/journal.pone.0053738 23301102 PMC3536740

[r9] Gibbs SG Green CF Tarwater PM Mota LC Mena KD Scarpino PV 2006 Isolation of antibiotic-resistant bacteria from the air plume downwind of a swine confined or concentrated animal feeding operation. Environ Health Perspect 114 1032 1037; 10.1289/ehp.8910 16835055 PMC1513331

[r10] Golding GR, Bryden L, Levett PN, McDonald RR, Wong A, Wylie J (2010). Livestock-associated methicillin-resistant *Staphylococcus aureus* sequence type 398 in humans, Canada.. Emerg Infect Dis.

[r11] Graveland H, Wagenaar JA, Bergs K, Heesterbeek H, Heederik D. 2011 Persistence of livestock associated MRSA CC398 in humans is dependent on intensity of animal contact. PLoS One 6 2 e1683; 10.1371/journal.pone.0016830 PMC3036727 21347386

[r12] Graveland H Wagenaar JA Heesterbeek H Mevius D van Duijkeren E Heederik D 2010 Methicillin-resistant *Staphylococcus aureus* ST398 in veal calf farming: human MRSA carriage related with animal antimicrobial usage and farm hygiene. PLoS One 5 6 e10990; 10.1371/journal.pone.0010990 20544020 PMC2882326

[r13] Harrison EM, Paterson GK, Holden MT, Larsen J, Stegger M, Larsen AR (2013). Whole genome sequencing identifies zoonotic transmission of MRSA isolates with the novel *mecA* homologue *mecC*.. EMBO Mol Med.

[r14] Hartmeyer GN, Gahrn-Hansen B, Skov RL, Kolmos HJ (2010). Pig-associated methicillin-resistant *Staphylococcus aureus*: family transmission and severe pneumonia in a newborn.. Scand J Infect Dis.

[r15] Khanna T, Friendship R, Dewey C, Weese JS (2008). Methicillin resistant *Staphylococcus aureus* colonization in pigs and pig farmers.. Vet Microbiol.

[r16] Klevens RM, Morrison MA, Fridkin SK, Reingold A, Petit S, Gershman K (2006). Community-associated methicillin-resistant *Staphylococcus aureus* and healthcare risk factors.. Emerg Infect Dis.

[r17] Lee BY, Singh A, David MZ, Bartsch SM, Slayton RB, Huang SS (2013). The economic burden of community-associated methicillin-resistant *Staphylococcus aureus* (CA-MRSA).. Clin Microbiol Infect.

[r18] Lewis HC, Molbak K, Reese C, Aarestrup FM, Selchau M, Sorum M (2008). Pigs as source of methicillin-resistant *Staphylococcus aureus* CC398 infections in humans, Denmark.. Emerg Infect Dis.

[r19] Lina G, Piemont Y, Godail-Gamot F, Bes M, Peter MO, Gauduchon V (1999). Involvement of Panton-Valentine leukocidin-producing *Staphylococcus aureus* in primary skin infections and pneumonia.. Clin Infect Dis.

[r20] McCarthy AJ, Witney AA, Gould KA, Moodley A, Guardabassi L, Voss A (2011). The distribution of mobile genetic elements (MGEs) in MRSA CC398 is associated with both host and country.. Genome Biol Evol.

[r21] Molla B, Byrne M, Abley M, Mathews J, Jackson CR, Fedorka-Cray P (2012). Epidemiology and genotypic characteristics of methicillin-resistant *Staphylococcus aureus* strains of porcine origin.. J Clin Microbiol.

[r22] Monaco M Pedroni P Sanchini A Bonomini A Indelicato A Pantosti A 2013 Livestock-associated methicillin-resistant *Staphylococcus aureus* responsible for human colonization and infection in an area of Italy with high density of pig farming. BMC Infect Dis 13 258; 10.1186/1471-2334-13-258 23731504 PMC3679754

[r23] Monecke S Coombs G Shore AC Coleman DC Akpaka P Borg M 2011 A field guide to pandemic, epidemic and sporadic clones of methicillin-resistant *Staphylococcus aureus.* PLoS One 6 4 e17936; 10.1371/journal.pone.0017936 21494333 PMC3071808

[r24] Morrison MA Hageman JC Klevens RM 2006 Case definition for community-associated methicillin-resistant *Staphylococcus aureus.* J Hosp Infect 62 2 241; 10.1016/j.jhin.2005.07.011 16289455

[r25] Oakes JM (2006). Commentary: advancing neighbourhood-effects research—selection, inferential support, and structural confounding.. Int J Epidemiol.

[r26] Osadebe LU, Hanson B, Smith TC, Heimer R (2013). Prevalence and characteristics of *Staphylococcus aureus* in Connecticut swine and swine farmers.. Zoonoses Public Health.

[r27] Pennsylvania Act 38 of 2005. (2005).

[r28] Price LB Stegger M Hasman H Aziz M Larsen J Andersen PS 2012 *Staphylococcus aureus* CC398: host adaptation and emergence of methicillin resistance in livestock. mBio 3 1 e00305-11; 10.1128/mBio.00305-11 22354957 PMC3280451

[r29] Rinsky JL Nadimpalli M Wing S Hall D Baron D Price LB 2013 Livestock-associated methicillin and multidrug resistant *Staphylococcus aureus* is present among industrial, not antibiotic-free livestock operation workers in North Carolina. PLoS One 8 7 e67641; 10.1371/journal.pone.0067641 23844044 PMC3699663

[r30] Salmenlinna S, Lyytikainen O, Vainio A, Myllyniemi AL, Raulo S, Kanerva M (2010). Human cases of methicillin-resistant *Staphylococcus aureus* CC398, Finland.. Emerg Infect Dis.

[r31] Schulz J, Friese A, Klees S, Tenhagen BA, Fetsch A, Rosler U (2012). Longitudinal study of the contamination of air and of soil surfaces in the vicinity of pig barns by livestock-associated methicillin-resistant *Staphylococcus aureus.*. Appl Environ Microbiol.

[r32] Shopsin B, Gomez M, Montgomery SO, Smith DH, Waddington M, Dodge DE (1999). Evaluation of protein A gene polymorphic region DNA sequencing for typing of *Staphylococcus aureus* strains.. J Clin Microbiol.

[r33] Smith TC Gebreyes WA Abley MJ Harper AL Forshey BM Male MJ 2013 Methicillin-resistant *Staphylococcus aureus* in pigs and farm workers on conventional and antibiotic-free swine farms in the USA. PLoS One 8 5 e63704; 10.1371/journal.pone.0063704 23667659 PMC3646818

[r34] Smith TC Male MJ Harper AL Kroeger JS Tinkler GP Moritz ED 2009 Methicillin-resistant *Staphylococcus aureus* (MRSA) strain ST398 is present in midwestern U.S. swine and swine workers. PLoS One 4 1 e4258; 10.1371/journal.pone.0004258 19145257 PMC2626282

[r35] Sunde M, Tharaldsen H, Marstein L, Haugum M, Norstrom M, Jacobsen T (2011). Detection of methicillin-resistant *Staphylococcus aureus* sequence type 8 in pigs, production environment, and human beings.. J Vet Diagn Invest.

[r36] Sung JM, Lloyd DH, Lindsay JA (2008). *Staphylococcus aureus* host specificity: comparative genomics of human versus animal isolates by multi-strain microarray.. Microbiology.

[r37] Tattevin P, Schwartz BS, Graber CJ, Volinski J, Bhukhen A, Bhukhen A (2012). Concurrent epidemics of skin and soft tissue infection and bloodstream infection due to community-associated methicillin-resistant *Staphylococcus aureus.*. Clin Infect Dis.

[r38] U.S. Environmental Protection Agency. (2012). Regulatory Definitions of Large CAFOs, Medium CAFO, and Small CAFOs.. http://www.epa.gov/npdes/pubs/sector_table.pdf.

[r39] van Cleef BA, Graveland H, Haenen AP, van de Giessen AW, Heederik D, Wagenaar JA (2011). Persistence of livestock-associated methicillin-resistant *Staphylococcus aureus* in field workers after short-term occupational exposure to pigs and veal calves.. J Clin Microbiol.

[r40] Verkaik NJ, Benard M, Boelens HA, de Vogel CP, Nouwen JL, Verbrugh HA (2011). Immune evasion cluster-positive bacteriophages are highly prevalent among human *Staphylococcus aureus* strains, but they are not essential in the first stages of nasal colonization.. Clin Microbiol Infect.

[r41] Vittinghoff E, Glidden D, Shiboski S, McCulloch C. (2005).

[r42] Voss A, Loeffen F, Bakker J, Klaassen C, Wulf M (2005). Methicillin-resistant *Staphylococcus aureus* in pig farming.. Emerg Infect Dis.

[r43] Waters AE, Contente-Cuomo T, Buchhagen J, Liu CM, Watson L, Pearce K (2011). Multidrug-Resistant *Staphylococcus aureus* in US Meat and Poultry.. Clin Infect Dis.

[r44] Wulf MW, Verduin CM, van Nes A, Huijsdens X, Voss A (2012). Infection and colonization with methicillin resistant *Staphylococcus aureus* ST398 versus other MRSA in an area with a high density of pig farms.. Eur J Clin Microbiol Infect Dis.

